# Atrio-Ventricular Dyssynchrony After Cardiac Resynchronization Therapy: An Unusual Contributor to Heart Failure Symptoms

**DOI:** 10.7759/cureus.35661

**Published:** 2023-03-01

**Authors:** Katta Lavanya, Okechukwu N Mgbemena, Stephen G Keim

**Affiliations:** 1 Cardiology, University of Florida College of Medicine – Jacksonville, Jacksonville, USA; 2 Electrophysiology, University of Florida College of Medicine – Jacksonville, Jacksonville, USA

**Keywords:** a and e wave in echo, echocardiogram (echo), interventricular dyssynchrony, av resynchrony, cardiac resynchronization therapy (crt)

## Abstract

Cardiac resynchronization therapy (CRT) is the mainstay for the management of systolic heart failure with LVEF <35% and evidence of dyssynchrony despite optimal medical therapy. After CRT placement, persistent dyssynchronization is possible and can contribute to heart failure symptoms despite a well-functioning CRT device. Echo-guided imaging can be beneficial for the optimization of CRT in selected patients who have evidence of continued dyssynchrony despite a well-functioning CRT device.

## Introduction

Cardiac resynchronization therapy (CRT) is the standard of care for refractory systolic heart failure despite optimal medical therapy in patients with evidence of dyssynchrony on the electrocardiogram (EKG) [1,2]. Currently, CRT is indicated for patients with systolic heart failure (LVEF < 35%), left bundle branch block (LBBB), and QRS duration >130 ms [3,4]. There is also a recommendation for CRT in patients with non-LBBB patterns with QRS > 150 ms and patients with expected pacing >40% of cardiac activity with LVEF <35-40% [5-7]. When patients receive CRT, a lead is placed in the right atrium, the right ventricle (RV), and in a branch of the coronary sinus, abutting the lateral wall of the left ventricle (LV) [8]. This configuration aims to restore intra-ventricular synchrony by activating electrical depolarization and subsequent mechanical contraction of the walls of the LV at the same time [9]. This is known as intra-ventricular re-synchronization. In addition to intra-ventricular resynchronization, the CRT device can also be optimized for atrio-ventricular (AV) and interventricular synchrony [10]. In atrio-ventricular synchrony, the device is programmed in such a way to allow complete emptying of the blood from the atrium into the ventricle before ventricular depolarization/contraction. Lastly, CRT can be optimized for interventricular synchrony - during which an optimal setting allows the maximal cardiac output of both RV and LV [11-13]. When any of these three synchronization axes is suboptimal, the associated hemodynamic consequence can contribute to heart failure symptoms. In this case report, we present this unusual contributor to heart failure symptoms in a patient with adequate intra-ventricular resynchronization but with persistent AV dyssynchrony.

## Case presentation

A 60-year-old female presents to the emergency department with an acute exacerbation of systolic heart failure. She has a past medical history of Marfan syndrome associated with abdominal aortic aneurysm (AAA) status post open repair with a Dacron graft, papillary thyroid carcinoma status post resection, aortic root dilation status post valve-sparing aortic root reconstruction, severe scoliosis with restrictive lung disease, diabetes, and anemia. After her valve-sparing aortic root reconstruction, the patient developed symptomatic bradycardia, which was treated with CRT placement. She also has a history of non-ischemic cardiomyopathy with an ejection fraction of 35-40% (NYHA class 3) and was on goal-directed medical therapy with carvedilol, valsartan, spironolactone, and empagliflozin. Although our patient was adherent to her medications, salt, and water restrictions, she continued to have frequent heart failure hospitalizations.

Investigations

**Figure 1:**
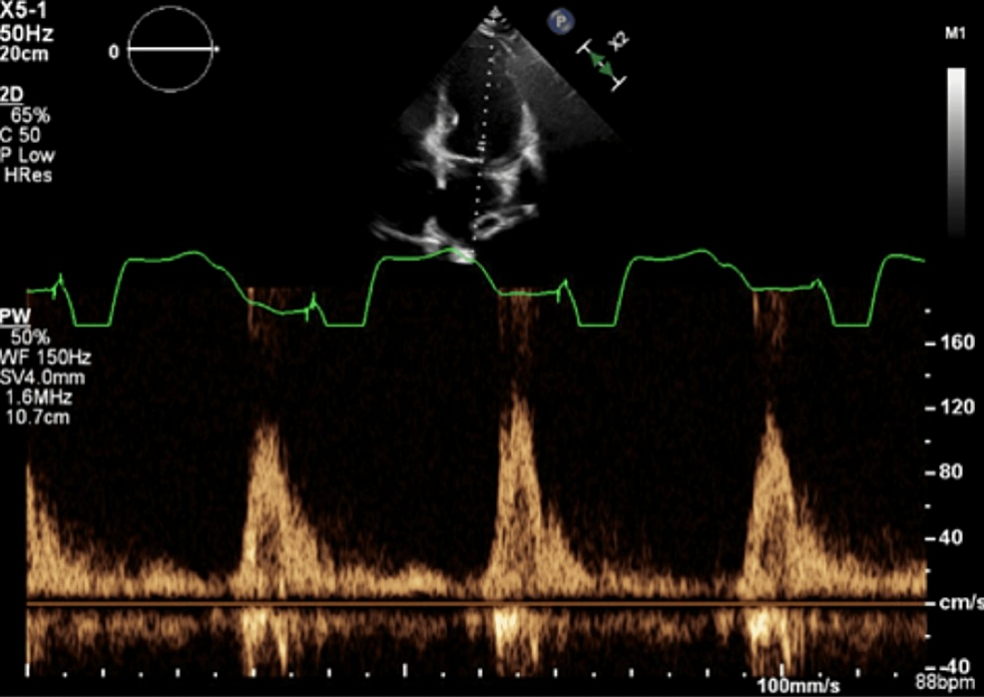
The mitral valve inflow pattern showed a fusion of E and A waves.

On physical examination, she had weight gain, bilateral leg edema, dyspnea on exertion, and orthopnea consistent with heart failure exacerbation. A chest X-ray demonstrated severe cardiomegaly with lung findings suggestive of pulmonary edema. She has an atrial sensed-v-paced rhythm on the EKG, and the echocardiogram was consistent with left ventricular systolic dysfunction - LVEF estimated at 15-20% and QRS duration was >130 ms. There was septal wall motion abnormality consistent with conduction abnormality and moderate aortic and tricuspid regurgitations, but these were unchanged compared to the prior echo six months ago. The mitral valve inflow pattern showed a fusion of E and A waves (Figure 1).

Differential diagnosis

The patient’s presentation was consistent with heart failure exacerbation given the presence of multiple FRAMINGHAM criteria, NYHA class 4 and elevated BNP of 8,098 PG/ML. The exacerbating factors were not immediately apparent as the patient had been consistent with salt and water restrictions and was adherent to her goal-directed medical therapies. There were no signs of infection, and her thyroid function was normal. There was no suspicion of illicit drug abuse. Due to the fusion of E and A waves on her mitral inflow doppler, there was a suspicion of atrio-ventricular dyssynchrony as a possible contributor to her persistent heart failure symptoms.

Management

**Figure 2:**
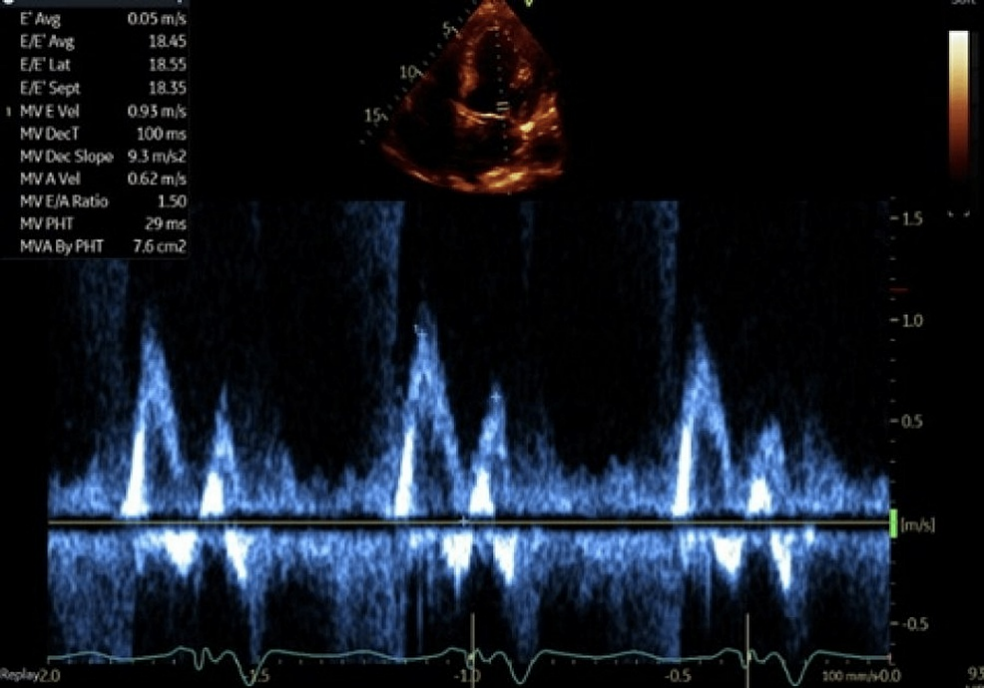
After the change in settings, there was emergence of a distinct A wave, separate from the E wave.

In addition to intravenous diuresis and optimization of medical therapy, the patient's CRT device was interrogated for AV dyssynchrony due to the fusion of E and A waves in her mitral inflow pattern. The left ventricular lead was pacing from LVRing2>>can. The mitral inflow pattern was suggestive of an AV delay causing atrio-ventricular dyssynchrony. To improve synchronization, simultaneous adjustments were made to the AV interval while monitoring the patient's mitral inflow pattern. The paced AV delay was shortened to 60-120 ms (from 170 ms), and the sensed AV delay was shortened to 50-100 ms (from 120 ms). After the change in settings, there was the emergence of a distinct A wave, separate from the E wave (Figure 2). Distinct A and E waves suggest improved atrio-ventricular synchrony, and the patient reported immediate symptomatic improvement after adjustment.

Follow-up

After echo-guided optimization, the patient reported symptomatic improvement. She was also given heart failure-specific diet counseling, and her GDMT was continued. She was subsequently discharged on hospital day 3.

## Discussion

**Figure 3:**
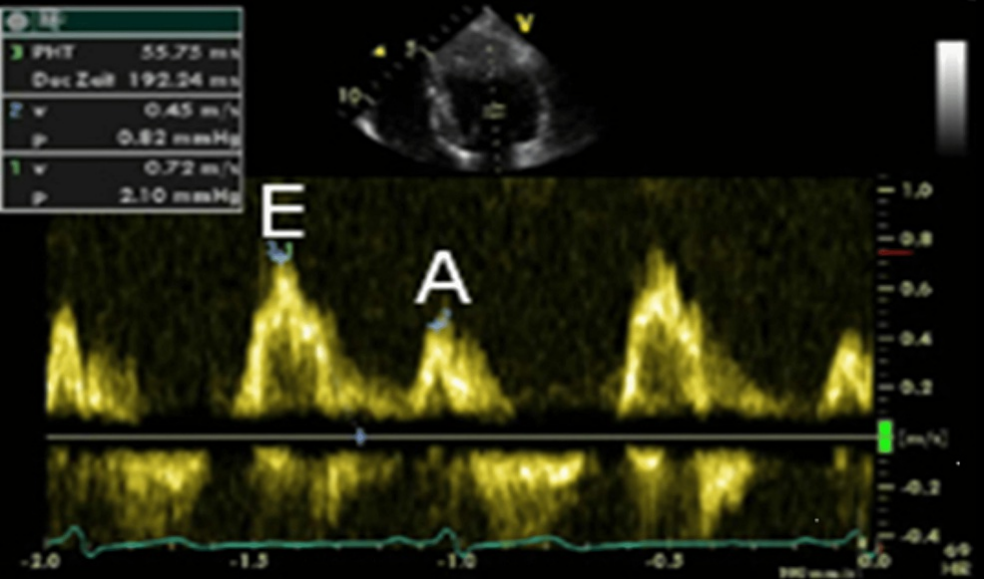
Normal mitral inflow velocity profile showing distinct E and A waves. The E wave is greater than A wave.

CRT is the standard of care for patients with worsening systolic heart failure despite GDMT and in whom there is evidence of dyssynchrony. Currently, CRT is indicated for patients with systolic heart failure (LVEF < 35%), left bundle branch block (LBBB), and QRS duration > 130 ms (class 1 according to ACCHRS) [14]. There is also a recommendation for CRT in patients with non-LBBB patterns with QRS > 150 ms and patients with expected pacing >40% of cardiac activity and LVEF ≤35-40% [15]. Early randomized controlled trials suggest improvements in heart failure hospitalizations and mortality in patients with NYHA III-IV heart failure, EF ≤35%, and QRS duration over 120 ms. After CRT placement, most patients are resynchronized using standard settings rather than imaging-guided resynchronization [16]. Echo-guided optimization of CRT remains controversial as the data supporting it remains inconclusive [17]. It is thought that the data supporting echo-guided optimization is inconclusive because of poor patient selection [17]. Indeed, there has been reported improvement after echo-guided optimization of CRT in select patients, especially in those without any other known cause of frequent heart failure hospitalizations [18]. The CRT device resynchronizes in three axes - AV, intraventricular, and interventricular resynchronizations. AV synchronization is related to optimal timing for atrial and ventricular pacing and is evaluated with a pulse doppler of the mitral inflow. A normal mitral inflow has distinct E and A waves (Figure 3). The absence or interruption of the atrial component of the mitral inflow is suggestive of suboptimal CRT programming.

**Figure 4:**
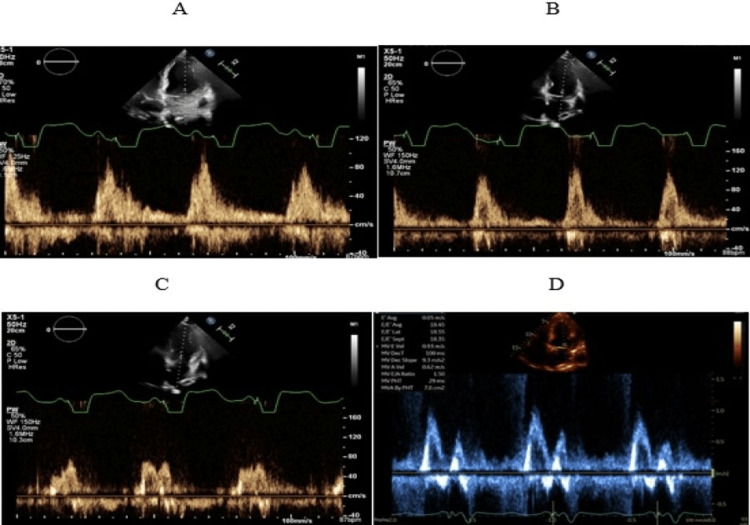
Showing AV delay of 170 milliseconds in (A), 150 milliseconds in (B), 120 milliseconds in (C), 60 milliseconds in (D)

The fusion of the A and E waves suggests that the AV delay is too long [19]. An abnormally long AV delay can result in diastolic mitral regurgitation, suboptimal LV preload, or may even allow native LV conduction, which defeats the purpose of CRT. On the other hand, the interruption of the A wave by ventricular contraction suggests an abnormally short AV delay. When AV dyssynchrony is present, the CRT benefit can be optimized through echo-guided adjustments to pacing parameters. Our patient had a prolonged AV delay resulting in the fusion of the E and A waves, which improved with the sequential decrement of the AV delay (Figure 4).

Interventricular synchrony is related to the optimization of right and left ventricular output. When there is interventricular dyssynchrony, there is a time delay between the onset of right and left ventricular output of more than 40 ms. To achieve interventricular optimization, the time to onset of flow in the RVOT and LVOT is set to be within 40 ms. Finally, intraventricular synchrony is related to the simultaneous activation of the septal and posterior walls of the left ventricle. Intraventricular dyssynchrony is suggested when the septal and posterior walls contract more than 130 ms apart. To improve intraventricular dyssynchrony, the pacing intervals/location of the pacing lead on the septal and posterior walls are adjusted until the difference in wall activation is less than 70-80 ms [19]. Table 1 summarizes echo-guided CRT optimization, echo modality, parameters, and time intervals [17-19].

**Table 1:** Echo modality, parameters and time intervals.

Echo modality	Parameter	Cut off time in milliseconds
M mode	Septal to posterior wall motion delay	>130 ms
Pulse wave (PW) Doppler echocardiography	Time difference between the onset of flow in the right and left ventricular outflow tracts	>40 ms
Tissue Doppler imaging echocardiography (TDI)	Time difference between opposing LV walls	>65 ms
Speckle tracking	Time difference between maximal thickening of the anteroseptal and posterior wall	>130 ms
Cross-correlation	Analysis of TDI curves, uses time difference of maximal peaks and integrates the whole shape of the velocity curve using cross-correlation spectrum	

## Conclusions

Dyssynchrony of the CRT device can contribute to heart failure exacerbations and symptoms. In selected patients without any other discernible etiology for heart failure exacerbation, echo-guided optimization of CRT may be beneficial if there is evidence of dyssynchrony. In these patients, careful examination of M-mode, pulse-wave/tissue doppler, speckle tracking, and cross-correlation waveforms may elucidate the need for echo-guided optimization.
